# Cross-Representational Interactions: Interface and Overlap Mechanisms

**DOI:** 10.3389/fpsyg.2016.02028

**Published:** 2017-01-04

**Authors:** Andriy Myachykov, Ashley J. Chapman, Martin H. Fischer

**Affiliations:** ^1^Department of Psychology, Northumbria UniversityNewcastle-upon-Tyne, UK; ^2^Centre for Cognition and Decision Making, National Research University Higher School of EconomicsMoscow, Russia; ^3^Department of Psychology, Northumbria UniversityNewcastle-upon-Tyne, UK; ^4^Division of Cognitive Science, University of PotsdamPotsdam, Germany

**Keywords:** representation, cross-representational interaction, simulation, embodiment, grounded cognition

## Abstract

A crucial question facing cognitive science concerns the nature of conceptual representations as well as the constraints on the interactions between them. One specific question we address in this paper is what makes cross-representational interplay possible? We offer two distinct theoretical scenarios: according to the first scenario, co-activated knowledge representations interact with the help of an interface established between them via congruent activation in a mediating third-party general cognitive mechanism, e.g., attention. According to the second scenario, co-activated knowledge representations interact due to an overlap between their features, for example when they share a magnitude component. First, we make a case for cross-representational interplay based on grounded and situated theories of cognition. Second, we discuss interface-based interactions between distinct (i.e., non-overlapping) knowledge representations. Third, we discuss how co-activated representations may share their architecture via partial overlap. Finally, we outline constraints regarding the flexibility of these proposed mechanisms.

A counterargument to this modular view is offered by embodied, grounded, or situated approaches to cognition, suggesting instead that conceptual knowledge is tightly integrated with the modal systems (Barsalou, 1999; Pulvermüller, 1999; Vigliocco et al., 2004; Myachykov et al., 2014). In this view, sensorimotor experiences form a constitutive part of semantic knowledge. While the debate continues (cf. Meteyard et al., 2012), here we follow embodied theories and discuss cognition as *simulation*, or “the re-enactment of perceptual, motor, and introspective states acquired during experience with the world, body, and mind” (Barsalou, 2009, p. 1281). This approach allows us to advance specific hypotheses regarding (1) oﬄine and online properties of co-activated conceptual representations as well as (2) the diagnostic features for the distinct interactive contexts.

Recent research into the cognitive representations of *apparently abstract*^1^ concepts is consistent with embodied approach: for example, representations of the concepts denoting *emotional valence* (Foroni and Semin, 2009), *time* (Bottini et al., 2015), and *number* (Myachykov et al., 2016) were shown to be intimately linked with the perceptual experiences associated with their acquisition and use. Supporting this claim, several studies demonstrated that regular *spatial-conceptual mappings* accompany processing of words or symbols with numerical (Fischer, 2003; Fischer et al., 2004), spatial (Richardson et al., 2003), emotional (Meier and Robinson, 2004), and temporal (Núñez and Cooperrider, 2013) semantics. For example, people map positive emotions onto upper space and negative emotions onto lower space (e.g., Meier and Robinson, 2004). Similarly, in the temporal domain, the past is associated with left space and the future – with right space (Núñez and Cooperrider, 2013). Finally, in the number domain, small magnitudes map onto left space and large magnitudes – onto right space (Fischer and Shaki, 2014, for review).

These and similar spatial associations provide evidence for the role of sensorimotor systems in activation of *single* concepts (i.e., numbers, space, valence, and time). However, given the complexity and richness of the sensorimotor contexts that typically accompany cognitive processes and the pervasive involvement of modal systems in abstract thought, it is plausible to assume that *co-activated concepts* stemming from distinct knowledge domains would also rely on embodied principles in their *interactions* – either by means of a shared general cognitive mechanism or by sharing aspects of their mental representations. Below, we offer a brief sketch of a theoretical account that describes a dual-route proposal for cross-representational interactions. Specifically, we argue that co-activated representations interact either via an *online interface* established by a general cognitive system (e.g., attention or memory) or by means of a more permanent *representational overlap*; that is, by virtue of two or more representations sharing parts of their core architecture (e.g., magnitude, affect, or sequencing).

Theories of knowledge often define “conceptual representation” as a combination of its core (or permanent) and situated (or online) features (Wilson, 2002; Myachykov et al., 2014). This distinction is important for our argument: we propose that interface-based interactions are limited to online or situated contexts while overlap between representations is a feature of permanently stored representations. Although, it is notoriously difficult to trace oﬄine their oﬄine features, one can document the differences between different *online* renditions of the same *oﬄine* representation. This can be illustrated with *the set effect* in Dehaene et al. (1993, Experiment 3): while oﬄine representations for numbers 4 or 5 may be insensitive to any spatial-numerical mappings, their activation is accompanied by different *situated* spatial mappings depending on the set, within which they are activated. Namely, 4 and 5 show rightward mapping, associated with larger numbers, when activated in the set 0–5 and leftward mapping, typical for small numbers, – when activated in the set 4–9. In other words, while horizontality may be an off-line feature for both 4 and 5 its exact online displacement can be either left- or right-orienting (cf. Chiou et al., 2009 for another example of a similarly flexible mapping mechanism).

## Representational Interface: Interactions Between (Relatively) Unrelated Representations

**FIGURE 1:**
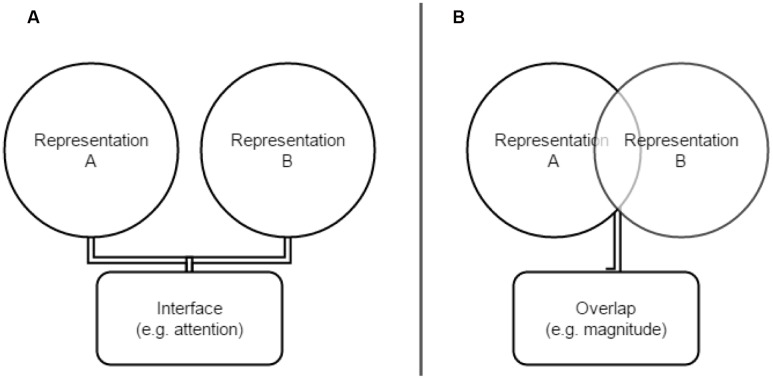
**(A)** Interface and **(B)** overlap interactions in co-activated representations.

When two conceptual representations become co-activated, they may interact even if their individual architectures share little common ground. This interaction is supported by simultaneously engaging a third party component that acts as a mediator, or *an interface* (**Figure 1A**). As noted above, consistent spatial conceptual mappings have been documented for several *distinct* knowledge domains (e.g., Cappelletti et al., 2009; Holmes and Lourenco, 2011; Bonato et al., 2012; Hayashi et al., 2013; Lachmair et al., 2014b; Santiago and Lakens, 2015; Winter et al., 2015). Importantly, these and similar findings are not epiphenomenal: systematic and largely automatic shifts of covert spatial attention were shown to accompany these spatial-conceptual mappings, leading to cross-domain priming and thereby indicating functional benefits of establishing the interface. This is typically done by using secondary tasks that accompany processing of apparently abstract concepts (e.g., visual probe detection tasks) co-occurring with or following, a word processing task (e.g., Richardson et al., 2003). We hypothesize that, when co-activated, concepts known to carry similar spatial mappings regularly interface via a shared attentional system, thereby leading to more efficient sensorimotor mappings (cf. also Rizzolatti et al., 1994).

Although, there is a distinct lack of studies investigating the role of attention in cross-representational interplay, a number of existing reports provide initial evidence supporting our claim. For example, in experiments described in Lachmair et al. (2014a,b) participants were faster to signal lexical decisions for upward-orienting nouns (e.g., *sun*) or verbs (e.g., *rise*) following upward-orienting number prime (e.g., 9). According to the interface scenario proposed here, this *priming effect* reflects activation of the attentional interface shared by the two distinct conceptual representations. In effect, activation of the first stimulus (the number) established a cue (Posner, 1980) biasing attention toward the upper space. Because the subsequently presented word also has the capacity to cue attention toward upper space, its processing was facilitated.

We followed a similar logic in our recent experiments where we presented participants with a task requiring them to process two different concepts *simultaneously* (Chapman et al., in preparation). Participants read an upward or downward orienting valence word (e.g., *hero/villain*) followed by an upward or downward orienting number (e.g., 1/9). This sequence was followed by the presentation of a spatially congruent or incongruent visual probe, for which participants provided manual RTs. Hence, the probe could be either fully or partially (in)congruent to the word’s and number’s biases. Crucially, participants had to maintain both the word and the number in their working memory at the point of the probe detection task as they were later asked about either the identity of the word or the number. Performance on the visual probe detection task, seemingly unrelated to either of the two co-activated representations, showed interaction between words’ and numbers’ spatial biases similar to the one observed in Lachmair et al. (2014a,b).

Together, these studies provide initial evidence for the role of the same attentional mechanism in supporting cross-representational interactions. These studies also lend support to the idea that (1) the attentional system underlying spatial biases in apparently abstract knowledge representations is relatively general and universal and (2) that any two simultaneously accessed representations that are known to induce spatial biases can interact via the shared attentional system (e.g., Posner and Fan, 2008).

The most important diagnostic criterion for interface-based cross-representational interactions is that the mediator or interface does not feature as a semantic component of the interacting concepts’ meanings. Assume that the word *hero* and the number *9* are interfaced by sharing the upward attentional bias. While their co-activation would be measurable as a consistent attentional shift, neither of these two concepts represents vertical attention as a feature of its core meaning. This diagnostic feature stems from the constraint on *when* an interface can be established. We argue that interface-based interactions are limited to *online* contexts within which two concepts appear in temporal or spatial contiguity. Stored representations by themselves do not make reference to this interface system.

Spatial biases and attention are arguably the most studied general cognitive mechanisms supporting situated representations. As a result, we use attention as the “showcase” for our interface proposal. It is, however, not the only candidate for the role of an interface. Under our proposal, *working memory* can also act as an interface allowing cross-representational interactions (cf. Barsalou, 2008). While traditional conceptualizations of working memory propose distinct mechanisms for activation of verbal and visuospatial content (Baddeley, 1986), current views postulate that the active components of long-term knowledge representations constitute the equivalent of “working memory” (Cowan, 2005) and thus support cross-domain interfacing. An example could be the current debate about the origin of spatial-numerical associations, where sequential ordering of concepts might lead to spatial biases (for review, Abrahamse et al., 2016). Generally speaking, we predict that two co-activated representations that (1) share activation context (relational memory) or (2) have a comparable activation status – will interact.

## Representational Overlap: Interactions Between Partially Related Representations

When co-activated representations are related via a shared architecture then such representations can interact by simultaneously activating the feature(s) that they *share permanently*, instead of looking for an interface as described above (e.g., Brunel et al., 2009; Rey et al., 2015; Riou et al., 2015). In comparison to the interface logic, (1) overlapping concepts simultaneously represent the overlapping feature as an integral semantic component and, as a result, (2) the overlap is pertinent for both online and off-line contexts. One instance of such overlap-based interaction occurs between the *size* (volume) of an afforded hand grip and the *size* (magnitude) of a numerical concept. Indeed, several studies confirmed that participant respond faster when the perceived object’s size corresponds to the perceived number’s magnitude (e.g., Andres et al., 2004, 2008; Lindemann et al., 2007; Moretto and Di Pellegrino, 2008; Ranzini et al., 2011, Gianelli et al., 2012; Namdar et al., 2014; Ranzini et al., 2015; van Dijck et al., 2015). These findings have been extended in other studies to include overlap between non-manipulable object size and numbers (Gabay et al., 2013), weight and numbers (Holmes and Lourenco, 2013), and sound volume and numbers (Heinemann et al., 2013).

Note that both *grip* and *number* specify magnitude in a general way (overlap) while *words of valence* and *numbers* (such as *hero* and *9*) do not directly represent spatial displacement (and thus require an attentional interface). One theory that details how numerical magnitude can be related to an associated property in another domain (e.g., grasp aperture) is *A Theory of Magnitude* (ATOM; Walsh, 2003, 2015). ATOM argues that number, duration, quantity, as well as other similar concepts share a generalized magnitude system. In other words, these representations have a partially *overlapping* architecture that potentiates interactions between them. ATOM makes a general and theoretically broad case for representational overlap with regard to magnitude and provides both behavioral and neuroanatomical evidence for the existence of representations with an overlapping magnitude component (but see Van Opstal and Verguts, 2013, for a critique of ATOM). As such, **Figure 1B** portrays a general case of ATOM as described by Walsh (2003, 2015).

Similarly, to the role of attention in the interface-based cross-representational interactions, *magnitude* acts as our showcase instance of *overlap-based* interactions. However, we believe this is not the only such mechanism. Two other mediators we propose as candidates for the cross-representational overlap are *sequencing* and *affect.* Although, there is limited research on how the brain’s sequencing system is involved during conceptual processing, recent neuroscientific research has attributed a special role to the cerebellum among other structures within a network supporting processes of chunking, patterning, and *sequencing* of different stimuli including sounds, words, and numbers (Dehaene et al., 2015). For example, the cerebellum has been implicated in mathematical cognition as well as in grammatical processes and music perception (Doya, 1999; Riva and Giorgi, 2000; Janata and Grafton, 2003; Wartenburger et al., 2003; Murdoch, 2010; Pliatsikas et al., 2014; Dehaene et al., 2015). We suggest that the brain network responsible for patterning, sequencing, and chunking can mediate cross-representational interaction effects for the concepts that rely heavily on hierarchical organization of linear sequences such as grammatical, arithmetic, and musical phrases (Scheepers et al., 2011; Van de Cavey and Hartsuiker, 2016; Van de Cavey et al., 2016).

Another possible overlap mediator is affect. Existing research (e.g., Berridge and Kringelbach, 2013) describes the affective system of the brain as a distinct network of cortical and subcortical (e.g., *amygdala*) structures involved during processing of emotional stimuli. This network’s components were previously shown to support processing of emotional words (Naccache et al., 2005) and emotional face judgements (Morris et al., 1998; Sheline et al., 2001; Pegna et al., 2005). One prediction would be that interactions between different valence projecting stimuli (e.g., words and faces) will lead to the registration of interaction effects similar to those described above for magnitude-based representations.

## A Case for Mixed Cases

The two-route mechanism described above suggests that while the overlapping feature is an integral part of a stored representation, the interface mediator *emerges* as a property of a situated context that supports interaction via simultaneously available reference to general cognitive mechanisms (e.g., attention) (cf. Jackendoff, 2002). Of course, one can assume that a reference or a command to interface with attentional networks when going online can be a permanent feature of a stored representation. In this case, this feature needs to be shared by the interacting representations. Finally, we postulate that interface and overlap mechanisms are distinct because one can test their activation differentially. Consider, for example, concepts denoting emotional valence may have *two* simultaneously available mechanisms allowing them to interact with other concepts: (1) an online interface mechanism based on attentional displacement and (2) an off-line overlap mechanism based on affect. Theoretically, these mechanisms can be activated simultaneously as well as independently. For example, interactions between numbers and valence words can be supported by an attentional interface while interactions between emotional faces and valence words can be supported by an affective overlap (cf. Holmes and Lourenco, 2009). In the same logic, time can also have different mapping mechanisms simultaneously available as the basis for interaction with other concepts: an attentional interface for the representation of the past *vs.* the future and magnitude-related overlap for the conceptualization of duration (e.g., Ma et al., 2012). Similarly, even the same interface type (e.g., spatial attention) can have simultaneously available mappings along different spatial dimensions. A recent study by Pitt and Casasanto (2016) provides an intriguing demonstration that, while the *polarity* of emotional valence (good vs. bad) tends to map vertically, its *magnitude* has a stronger representation in horizontal space. Papies (2013) used a similar approach in order to distinguish semantic and grounded/embodied features of food representations (cf. Freddi et al., 2016).

Further research is necessary in order to investigate the nature of these and similar representational pairs with flexible interaction systems. One particular question is whether the overlap and interface interaction systems are activated independently or whether the cognitive system habitually uses both interfaces at the same time, thus making the established interaction structure stronger. The examples from the spatial-numerical domain given last suggest that attentional interfaces are more rapidly established and modulated, and thus have a substantially faster time course of engagement when compared to the featural overlap mechanism: scanning number words from right to left (in Hebrew) or from left to right (in Russian) modulated the SNARC effect within seconds, while reading text with small and large numbers positioned in a SNARC-congruent vs. SNARC-incongruent way only gradually led to a modulation of the SNARC effect over several minutes. Other predictions stemming from our proposal concern the brain regions involved in setting up attentional vs. featural interfaces. Specifically, cross-representational interactions established via an attentional interface should be accompanied by cortical activations within well-known attentional networks (Fan et al., 2005) while interactions aided by featural interfaces should be less dependent on such focal activations and instead activate distributed brain areas that represent distinct knowledge domains as well as cerebellar structures.

Finally, with regard to the acquisition of semantic interfaces, one implication of our proposal is that, by means of a repeatedly shared attentional mechanism, any two seemingly distinct knowledge representations may eventually interact once they each have become associated with systematically overlapping sensory or motor features. Thus, a Hebbian learning mechanism will eventually transform the attentional interface into a structural overlap that supports efficient cognition (Pulvermüller, 2013).

## Conclusion

In this paper, we made a theoretical proposal for two distinct interaction systems for grounded representations. We suggest that representing and activating knowledge in distinct conceptual domains is based on sensorimotor simulation, and further suggest that representations from different domains can interact either via a third party online interface or by permanently sharing a feature allowing for a representational overlap. We argue that our proposal is supported by evidence from research on number processing, words of emotional valence, and conceptual and linguistic concepts of manipulable objects. We hope this short sketch of proposed mechanisms and their basic features will encourage future integrative research on cross-representational interaction mechanisms.

## Ethics Statement

The research referred to in this paper has been approved by the Ethics Committee of Psychology Department, Northumbria University. Participants provided informed written consent.

## Author Contributions

AM, AC, and MF have contributed equally to this submission.

## Conflict of Interest Statement

The authors declare that the research was conducted in the absence of any commercial or financial relationships that could be construed as a potential conflict of interest.

## Footnotes

1 Here, we use the term “apparently abstract” whenever we refer to concepts that are traditionally rendered *disembodied, experience-independent*, and/or *symbolic* (e.g., Dove, 2009; Chatterjee, 2010; Zwaan, 2014).
